# Sunitinib for adenocarcinoma of the rete testis: a case report

**DOI:** 10.3389/fonc.2024.1358133

**Published:** 2024-04-08

**Authors:** Kezhen Li, Di Chen, Mingdong He, Jun Yu, Hua Mi

**Affiliations:** Department of Urology, The First Affiliated Hospital of Guangxi Medical University, Nanning, Guangxi, China

**Keywords:** case report, sunitinib, AORT, diagnostic, targeted therapy

## Abstract

**Background:**

Adenocarcinoma of the rete testis (AORT) is an extremely rare and aggressive tumor with a poor prognosis. Its etiology and pathological characteristics have not been extensively studied, making accurate diagnosis and appropriate management challenging. AORT, an invasive testicular tumor with a mortality rate of 46%, treatment typically involves radical orchiectomy, retroperitoneal pelvic lymph node dissection (RPLND), adjuvant chemotherapy, and/or ongoing monitoring, but the response to conventional radiation and chemotherapy is limited. At present, no effective targeted therapy for AORT has been found.

**Case description:**

In this case report, we present the clinical scenario of a 50-year-old male patient initially diagnosed with a right testicular hydrocele, who subsequently underwent eversion of the parietal tunica vaginalis. Postoperative pathological analysis revealed metastatic clear cell renal cell carcinoma (ccRCC). PET/CT demonstrated findings suggestive of left renal upper pole carcinoma with involvement of the right scrotum, para-aortic region, bilateral iliac vessels, bilateral inguinal region, and multiple metastases. Sunitinib, a tyrosine kinase inhibitor, is commonly employed in the treatment of ccRCC. The patient underwent treatment with sunitinib for a duration of 20 months, resulting in the inactivation of multiple metastases. Following this, a radical orchiectomy was performed, and the postoperative pathology confirmed the presence of AORT. This article provides a comprehensive account of the patient's medical history, diagnostic process, treatment modalities, and subsequent follow-up observations.

**Conclusions:**

This case report highlights the successful use of targeted therapy with sunitinib in a patient with AORT. The patient showed a positive response to targeted therapy. This study not only provides a novel foundation for the treatment of AORT, but also offers valuable insights for future treatment strategies in managing this particular form of testicular cancer.

## Highlights

- The successful use of targeted therapy with sunitinib in a patient with AORT and multiple lymph node metastases. What is known and what is new?
- AORT, an invasive testicular tumor with a mortality rate of 46%, treatment typically involves radical orchiectomy, RPLND, adjuvant chemotherapy, and/or ongoing monitoring, but the response to conventional radiation and chemotherapy is limited.
- This AORT patient showed a positive response to sunitinib, leading to the inactivation of all lymph node metastatic lesions and subsequent curative surgery. What is the implication, and what should change now?
- The successful response to targeted therapy in this case highlights the potential of personalized treatment approaches for rare and aggressive tumors such as AORT. However, further studies are needed to evaluate the efficacy and optimal duration of targeted therapy in AORT.

## Introduction

Adenocarcinoma of the rete testis (AORT) is an extremely rare malignant tumor that arises from the rete testis, a network of tubules located in the mediastinum testis. It accounts for 1%-2% of all testicular tumors and has a poor prognosis due to its aggressive nature and limited treatment options (1). The etiology and pathological characteristics of AORT are not well understood, and its rarity often leads to misdiagnosis and delayed treatment. In this case report, we aim to provide a comprehensive overview of the diagnostic and therapeutic journey of a patient with AORT, highlighting the challenges faced and the successful outcome achieved through targeted therapy and curative surgery.

## Case presentation

A 50-year-old male patient presented to a local hospital on January, 2022, with a chief complaint of "enlarged right testicle for more than 10 years." The patient denies having a family medical history, psychosocial history, or genetic history. Physical examination revealed significant enlargement of the right testicle, which was soft in consistency without palpable nodules or tenderness. Ultrasound examination indicated testicular hydrocele. Initial diagnosis was right testicular hydrocele, and the patient underwent testicular tunica vaginalis inversion surgery. The surgical procedure for the patient went smoothly. However, pathology after testicular tunica vaginalis inversion surgery suggested a high possibility of metastatic clear cell renal cell carcinoma(ccRCC). The patient was subsequently referred to our hospital for further evaluation and management.

**Figure 1:**
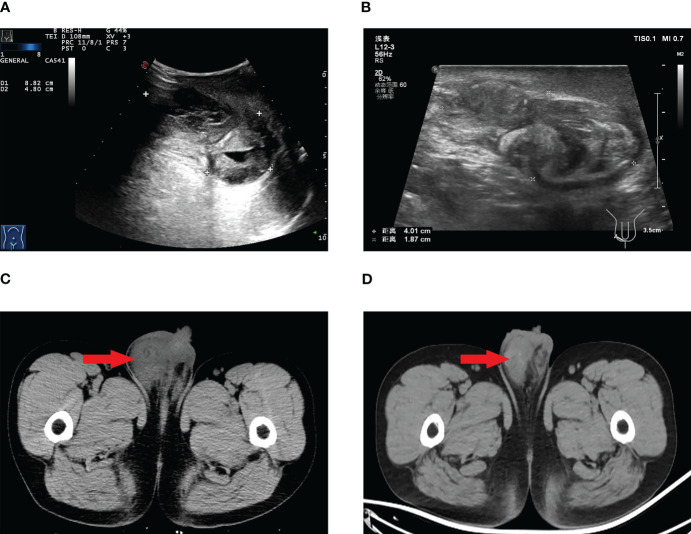
Scrotal ultrasound before targeted therapy **(A)**, scrotal ultrasound after targeted therapy **(B)**, scrotal CT before targeted therapy **(C)**, scrotal CT after targeted therapy **(D)**.

Laboratory investigations, including tumor marker analysis, did not show elevated levels of alpha-fetoprotein (AFP), lactate dehydrogenase (LDH), carbohydrate antigen 199 (CA199), carbohydrate antigen 125 (CA125), carcinoembryonic antigen (CEA), β-human chorionic gonadotropin(β-HCG), or prostate-specific antigen (PSA). Scrotal ultrasound and computed tomography (CT) scans revealed a mass in the right scrotum, raising suspicion of malignancy (**Figure 1**).

**Figure 2:**
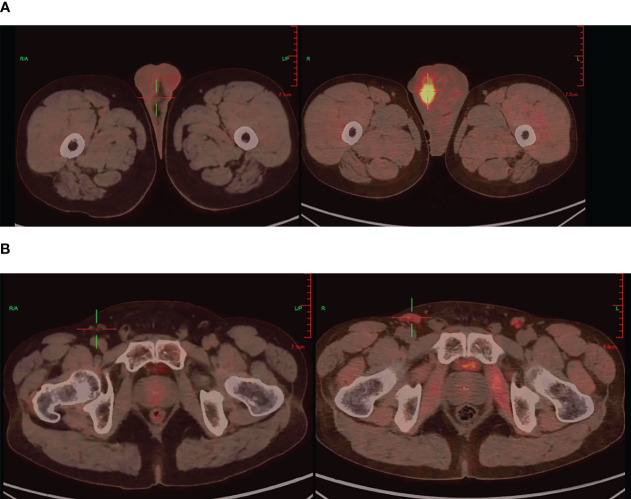
PET/CT imaging: Testicular before targeted therapy (right), after targeted therapy (left) **(A)**, Inguinal lymph nodes before targeted therapy (right), after targeted therapy (left) **(B)**.

Further imaging with positron emission tomography combined with computed tomography (PET/CT) demonstrated findings suggestive of left renal upper pole carcinoma with involvement of the right scrotum, para-aortic region, bilateral iliac vessels, bilateral inguinal region, and multiple metastases (**Figure 2**).

This patient and his family refused percutaneous renal biopsy or resection of the left kidney tumor. Based on the pathological results and imaging findings, the final diagnosis was determined as metastases ccRCC. After thorough communication with the patient and family, a decision was made to initiate targeted therapy with sunitinib, a tyrosine kinase inhibitor known to inhibit tumor growth and angiogenesis. The treatment regimen consisted of 4 weeks of medication followed by a 2-week drug-free period, and continued for 18 months.

During the course of targeted therapy, the patient underwent regular follow-up imaging studies to monitor the response to treatment. Gradual reduction in the size of the right testicular mass was observed on the imaging studies, indicating a positive response to sunitinib. A subsequent PET/CT scan revealed the following results after targeted therapy: A) The tumor activity in the right scrotal lesion was still present but showed improvement compared to previous scans; B) The previously identified metastatic lymph nodes showed no tumor activity; C) No new lesions were observed (**Figure 2**). These findings suggested a favorable response to targeted therapy, paving the way for curative surgery.

**Figure 3:**
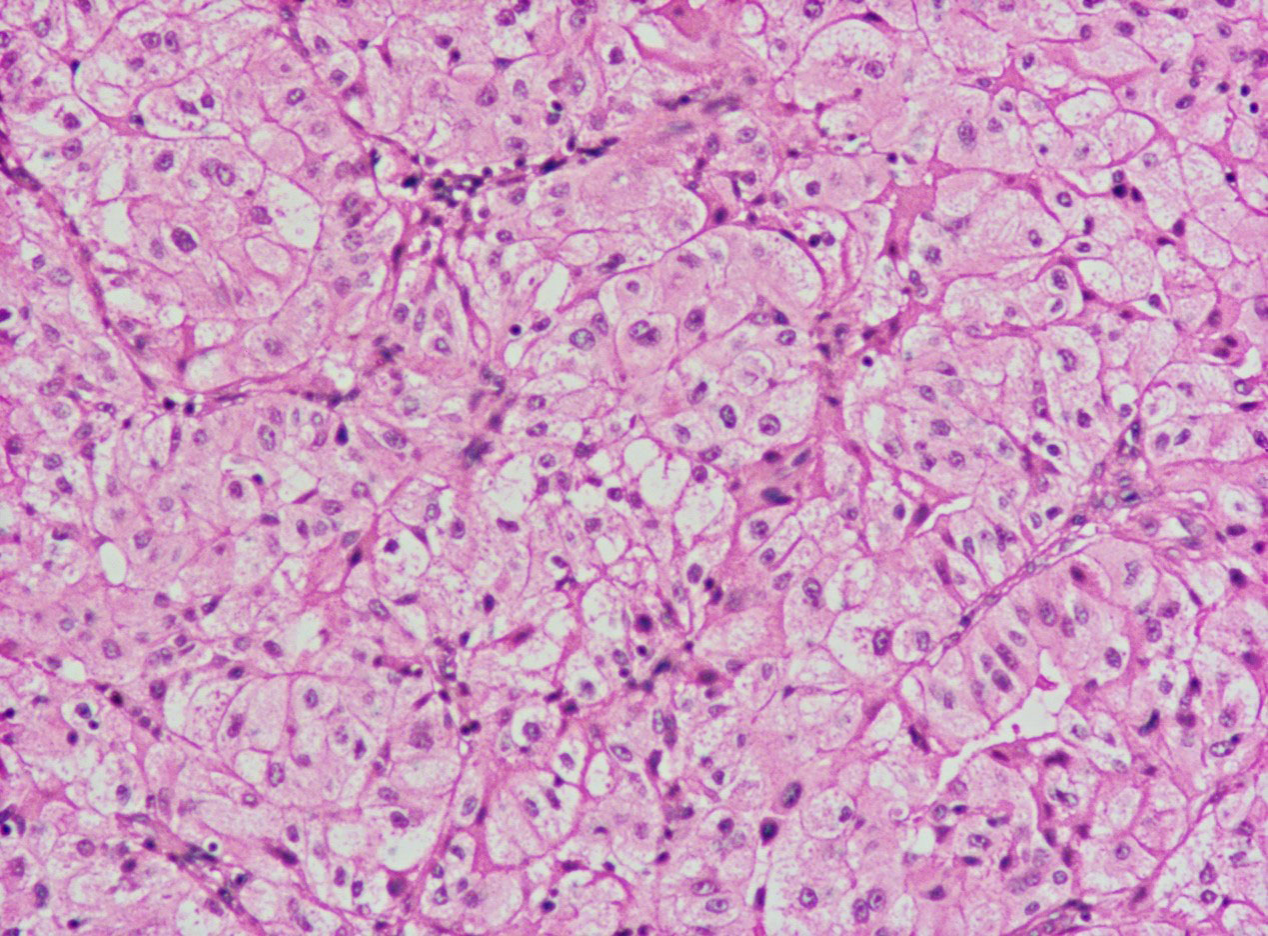
The visible component in the image resembles adenocarcinoma of the rete testis (AORT).

The patient discontinued sunitinib in August 2023 and underwent curative surgery for right testicular cancer in November 2023. The patient did not undergo kidney surgery because PET/CT indicated that the left renal lesion had disappeared. The postoperative pathological evaluation indicated: 1) Both tumors were of the same origin; 2) Considering the clinical and imaging findings, excluding metastasis from ccRCC, the diagnosis was determined as AORT (**Figure 3**). The timeline with relevant data as **Table 1**.

**Table 1:** Immunohistochemical staining results.

Immunohistochemical Characteristics of Adenocarcinoma of the Rete Testis	Result
EMA	+
Vimentin	+
PAX-8	+
PAX-2	–
CK7	–
CD10	–
HMB45	–
CAIX	+
Inhibin	–
CK5/6	–
CK-H	–
CD56	–
D2-40	–
CR	+
Ki-67#	+ 20%
CK8/18	+
S100	–
CD117	–
PLAP	–
CgA	–
Syn	–

## Discussion

AORT is an extremely rare disease, with fewer than 80 cases reported in the literature since its initial description in 1945 (2). The etiology and pathological characteristics of AORT remain poorly understood, making accurate diagnosis and appropriate management challenging. The majority of reported cases occur in Caucasian patients, and the tumor primarily affects middle-aged to elderly individuals (1).

The exact cause of AORT is still unclear, although some studies have proposed associations between tumor occurrence and various conditions and chemical exposures. These include hydrocele, chronic epididymitis or orchitis, testicular trauma, and exposure to certain chemicals such as asbestos and heavy metals (3–6). However, further research is needed to establish a definitive link between these factors and the development of AORT.

The diagnosis of AORT is often challenging due to its rarity and nonspecific clinical presentation. Patients may present with symptoms such as testicular enlargement, pain, or a palpable mass (2). However, these symptoms can also be seen in other testicular conditions, leading to misdiagnosis or delayed diagnosis.

Cases reported by Kitano et al. showed that serum CA 199 levels increase with the progression of AORT metastasis (7). There have also been reports of primary AORT associated with elevated serum CEA (8). However, as for AORT, there are currently no definitive tumor biomarkers. In this case, the patient’s AFP, β-HCG, CEA, CA 199, and other markers were within the normal range, with the only elevation being in serum ferritin. To date, there is no research demonstrating that serum ferritin can serve as a biomarker for AORT, and its role requires further investigation.

Imaging studies, including scrotal ultrasound and CT scans, play a crucial role in the initial evaluation of patients with suspected AORT. PET/CT scans are useful in identifying metastatic lesions and assessing treatment response.

The diagnosis of AORT is typically confirmed by postoperative pathology (9). Testicular gate localization, the transition from benign to malignant epithelium, and supportive immunostaining contribute to its accurate diagnosis (1). Research has shown that AE 1/AE 3 cytokeratin is the most consistent positive stain, followed by EMA,and vimentin (10, 11). In this case, the patient’s immunohistochemical results indicated EMA (+),and vimentin (+) (**Table 2**),consistent with the pathological diagnosis of AORT.

**Table 2:** The timeline with relevant data.

January 2022	Initial visit: Clinical symptoms: Swelling of the scrotum Physical examination: Marked enlargement of the right testicle Imaging findings: Ultrasound indicates hydrocele of the right testicle Diagnosis: Hydrocele of the right testicle Treatment: Testicular tunica vaginalis inversion surgery.
February 2022	Pathological examination: metastatic ccRCC. PET/CT: Left renal upper pole carcinoma with multiple metastases. Diagnosis: ccRCC with multiple metastases. Treatment: Sunitinib.
August 2023	Discontinuation of sunitinib. No significant discomfort observed during sunitinib treatment.
September 2023	CT: Decreased size of the right scrotal mass compared to previous examination.
October 2023	PET/CT: Decreased activity of the right scrotal tumor, no tumor activity observed in the left kidney, and no tumor activity observed in previously affected multiple lymph nodes.
November 2023	Treatment: Curative surgery for right testicular cancer. Pathological examination: AORT.

The management of AORT is complex and requires a multidisciplinary approach. Due to the rarity of the disease, there is no standard treatment protocol, and management decisions are often based on individual cases and expert opinions. Surgical resection, including radical orchiectomy, is the mainstay of treatment for localized disease. However, in cases of advanced or metastatic disease, systemic therapy options such as chemotherapy, targeted therapy, or immunotherapy may be considered.

AORT, a rare and invasive testicular tumor with a mortality rate of 46%, requires a multidisciplinary approach (1). Treatment typically involves radical orchiectomy, retroperitoneal pelvic lymph node dissection (RPLND), adjuvant chemotherapy, and/or ongoing monitoring, but the response to conventional radiation and chemotherapy is limited (2). In addition, AORT tends to metastasize to retroperitoneal lymph nodes, some cases undergoing RPLND have shown better outcomes, but this difference is not statistically significant (12). As far, it remains unclear whether RPLND changes patient outcomes. Due to the rarity of the disease, there is no standard treatment protocol. Paclitaxel, ifosfamide, and cisplatin are potential new drugs for treating AORT (13). Studies suggest that platinum-based chemotherapy may be effective against AORT, the patient with AORT who received bleomycin, etoposide, and platinum treatment had no further enlargement of metastatic lymph nodes after 7 months of discontinuing chemotherapy, and the patient remained alive at 20 months after discontinuation of chemotherapy (14).

In the case presented, the patient underwent targeted therapy with sunitinib, a tyrosine kinase inhibitor that has shown efficacy in the treatment of various solid tumors. Sunitinib inhibits multiple receptor tyrosine kinases, including vascular endothelial growth factor receptors (VEGFRs), platelet-derived growth factor receptors (PDGFRs), and stem cell factor receptor (KIT), thereby inhibiting tumor growth and angiogenesis (15). The patient showed a positive response to sunitinib, with significant reduction in the size of the testicular mass and complete inactivation of metastatic lymph nodes.

Analysis of existing data shows a median survival time of 33 months for patients with AORT. The 3-year and 5-year survival rates are 45% and 20%, respectively, with a 3-year survival rate of 18% for metastatic patients (12). In this case, the patient’s scrotal mass decreased after treatment with sunitinib, and the tumor activity in the metastatic lesions disappeared. Previous studies have shown that tumor confinement to the testicle is the most important prognostic factor for survival (12). The conditions for performing radical surgery for testicular tumors were created for the patient, and the patient did not experience any significant discomfort or dissatisfaction during the treatment process.

The successful response to targeted therapy in this case highlights the potential of personalized treatment approaches for rare and aggressive tumors such as AORT. Due to the inherent limitations of a case report, including potential biases and the lack of a comparison with a control group, the findings of this study have limited generalizability. Therefore, further studies are needed to evaluate the efficacy and optimal duration of targeted therapy in AORT. Additionally, the long-term outcomes and potential side effects of targeted therapy in AORT patients require careful monitoring and investigation.

## Conclusion

This case report highlights the successful use of targeted therapy with sunitinib in a patient with AORT and multiple lymph node metastases. The patient showed a positive response to targeted therapy, leading to the inactivation of all lymph node metastatic lesions and subsequent curative surgery. This study not only provides a novel foundation for the treatment of AORT, but also offers valuable insights for future diagnoses and treatment strategies in managing this particular form of testicular cancer.

## Data availability statement

The original contributions presented in the study are included in the article/supplementary material. Further inquiries can be directed to the corresponding author.

## Ethics statement

The studies involving humans were approved by First Affiliated Hospital of Guangxi Medical University ethics board. The studies were conducted in accordance with the local legislation and institutional requirements. The participants provided their written informed consent to participate in this study. Written informed consent was obtained from the individual(s) for the publication of any potentially identifiable images or data included in this article.

## Author contributions

KL: Writing – original draft, Writing – review & editing. DC: Writing – original draft, Writing – review & editing. MH: Writing – original draft, Writing – review & editing. JY: Writing – original draft, Writing – review & editing. HM: Writing – original draft, Writing – review & editing.
